# How, for whom, and in what contexts will artificial intelligence be adopted in pathology? A realist interview study

**DOI:** 10.1093/jamia/ocac254

**Published:** 2022-12-24

**Authors:** Henry King, Bethany Williams, Darren Treanor, Rebecca Randell

**Affiliations:** School of Medicine, University of Leeds, Leeds, UK; Department of Pathology, Leeds Teaching Hospitals NHS Trust, Leeds, UK; School of Medicine, University of Leeds, Leeds, UK; Department of Pathology, Leeds Teaching Hospitals NHS Trust, Leeds, UK; Department of Clinical Pathology, Linköping University, Linköping, Sweden; Department of Clinical and Experimental Medicine, Linköping University, Linköping, Sweden; Center for Medical Image Science and Visualization (CMIV), Linköping University, Linköping, Sweden; Faculty of Health Studies, University of Bradford, Bradford, UK; Wolfson Centre for Applied Health Research, Bradford, UK

**Keywords:** artificial intelligence, pathology, implementation, realist evaluation, qualitative research

## Abstract

**Objective:**

There is increasing interest in using artificial intelligence (AI) in pathology to improve accuracy and efficiency. Studies of clinicians’ perceptions of AI have found only moderate acceptability, suggesting further research is needed regarding integration into clinical practice. This study aimed to explore stakeholders’ theories concerning how and in what contexts AI is likely to become integrated into pathology.

**Materials and Methods:**

A literature review provided tentative theories that were revised through a realist interview study with 20 pathologists and 5 pathology trainees. Questions sought to elicit whether, and in what ways, the tentative theories fitted with interviewees’ perceptions and experiences. Analysis focused on identifying the contextual factors that may support or constrain uptake of AI in pathology.

**Results:**

Interviews highlighted the importance of trust in AI, with interviewees emphasizing evaluation and the opportunity for pathologists to become familiar with AI as means for establishing trust. Interviewees expressed a desire to be involved in design and implementation of AI tools, to ensure such tools address pressing needs, but needs vary by subspecialty. Workflow integration is desired but whether AI tools should work automatically will vary according to the task and the context.

**Conclusions:**

It must not be assumed that AI tools that provide benefit in one subspecialty will provide benefit in others. Pathologists should be involved in the decision to introduce AI, with opportunity to assess strengths and weaknesses. Further research is needed concerning the evidence required to satisfy pathologists regarding the benefits of AI.

## INTRODUCTION

Technological advances mean it is now possible to digitize pathology slides, creating the possibility for artificial intelligence (AI) to support pathologists’ work,^1^ a move for which there is enthusiasm.^2^ Recent reviews of studies of clinicians’ perceptions of AI describe positive attitudes regarding the potential for improved diagnostic accuracy, fewer errors, and more efficient workflows.^3^ However, acceptability was moderate,^4^ with concerns about liability, reputational loss, lack of evidence of efficacy in clinical settings, and lack of explainability,^3^ as well as key themes of lack of trust in patient safety and technology maturity.^4^ Such concerns raise the question of what is needed for integration of AI into pathology.

An international survey of pathologists found generally positive attitudes towards AI, seeing it as a tool to improve workflow efficiency and quality assurance.^5^ However, there were concerns about job displacement and replacement and a perceived need for education and training. An international survey of pathologists who regularly analyze dermatopathology slides found respondents were generally optimistic about AI, but anticipated it being useful for narrow, specified tasks.^6^ A Dutch interview study found pathologists were cautiously positive about AI, emphasizing improved workflow efficiency through supporting simple, routine, or repetitive tasks, but also felt AI would not be relevant to all pathology subspecialties.^7^ Need for patient benefit and cost to be balanced was also acknowledged. However, no study has considered factors necessary for adoption of AI in pathology.

Given this gap, we sought to answer the question: how and in what contexts is AI likely to become integrated into pathology? To do this, we used realist methods, which offer a framework for eliciting, testing, and refining stakeholders’ theories of how an intervention works.^8^ We began by reviewing reports regarding the potential of AI in pathology to elicit stakeholders’ theories and then interviewed pathologists to refine these theories. Below we describe our methods and summarize the literature-based theories that informed the interview study. We then present our results. We conclude by discussing implications for designers, evaluators, and healthcare organizations.

## MATERIALS AND METHODS

AI is a complex intervention.^9^ Studying complex interventions requires a strong theoretical foundation.^10^ While theories may sometimes be considered abstract and irrelevant, the term can also be used to refer to practitioners’ ideas about how an intervention works.^11^ This is how the term is used in realist studies, where theories typically combine substantive theory and stakeholders’ theories derived from experience.^8^^,^^11^ Realist methods have been used for studying numerous complex interventions, including health information technology (HIT),^12^ and in both evaluation and design.^13^

For realists, interventions do not lead to outcomes; rather outcomes depend on how recipients respond to the intervention, which depends on context (see Box 1 for an explanation of how context is understood in realist studies). In realist studies, stakeholders’ theories are presented as Context Mechanism Outcome configurations (CMOcs), detailing how intervention components trigger responses in users (mechanisms) within particular contexts to generate outcomes, providing an understanding of what works, for whom, in what circumstances, and how.

Box 1. Context in realist studies

In realist studies, ‘context’ does not refer to just people or things; although it may include these, relevant contextual factors can be psychological, organizational, or technical forces.^14^ Context is made up of multiple layers, including characteristics and capacities of stakeholders; relationships between stakeholders; institutional settings into which the intervention is introduced; and the wider social, economic, and cultural setting of the intervention.^15^ In realist studies of HIT, relevant contextual factors identified have included resources, such as computing resources and support staff; users’ work routines, motivation, and confidence in HIT use; and the implementation process.^13^

Realist studies start with elicitation of stakeholders’ theories. This can be done through interviewing stakeholders, reviewing existing literature, identifying theories from the sociological or other literatures, or some combination of these approaches. We began with a literature review (see Box 2; fuller details are reported elsewhere^16^). From this, we developed tentative CMOcs (Table 1) and used interviews to refine them.

Box 2. Theory elicitation literature review

Method: ‘Theory elicitation’ phase of a realist review,^17^ to answer the question “What works, for whom, in what circumstances, and how to encourage uptake and impact of AI in pathology?”

Search strategy: Search 1—Studies, reports, and policy documents were sought from: arXiv.org (Cornell University) repository, Ovid MEDLINE^®^, and HMIC Health Management Information Consortium (Ovid). Searches were developed for the concepts: AI and Histopathology, with subject headings and free text words identified by an Information Specialist and project team members and further terms identified and tested from known relevant papers. Search results were limited to English-language publications published since 2000. Searches were peer-reviewed by a second Information Specialist. Search 2—An ‘opinion leader’ search sought reports discussing AI authored by Eric Topol in Ovid MEDLINE^®^, Sciences Citation Index (Clarivate Analytics Web of Science), and Emerging Sources Citation Index (Clarivate Analytics Web of Science). Searches were also undertaken of relevant websites, for example, the College of American Pathologists, the Digital Pathology Association, along with a number of Google searches. Additional papers were identified through personal recommendation and searching of reference lists of included papers.

Selection and appraisal of documents: Papers were screened for relevance to the review question, first by title and abstracts and then based on review of the full text.

Data extraction, analysis, and synthesis: Documents were entered into Nvivo 12. Sections of text were indexed in an iterative process, using a series of codes that evolved to represent topics relevant to the review question. Following the realist strategy, these codes sought to capture different contexts, mechanisms, and outcomes that could affect the introduction of AI in pathology. The coded data were used to produce narrative summaries of each of the identified contexts, mechanisms, and outcomes. The reviewers and other authors then discussed these narratives and translated them into CMO configurations. The documents said little about the mechanisms through which the outcomes were achieved so we also drew on substantive theories concerning the implementation of technology and complex interventions more generally to fill this gap in our understanding.

Results: The search identified a total of 1433 unique records, 101 of which were found to be relevant. The analysis suggested: benefits of AI will vary according to the size and nature of the pathology department’s workload and the extent to which pathologists work collaboratively, with specialist centers benefitting from reduced workload, rather than increased accuracy; as with other areas of healthcare,^3^ pathologist trust is essential for uptake of AI,^16^ although less so for simple quantitative tasks. Relevant theories suggested AI is more likely to be accepted if pathologists are able to ‘make sense’ of the technology, engaged in the adoption process, supported in adapting their work processes, and can identify potential benefits to its introduction.^18–20^

**Table 1.** Context–Mechanism–Outcome configurations from the literature review

	Who	Context	+	Mechanism		=	Outcome
				Resource	Response		
#1		Iterative design process with early user involvement Pathologists involved in decision to implement AI in their department and are given the opportunity to regularly feed into reviews of its benefits and costs	+	AI undertakes quantitative tasks High usability Integration into workflow	Pathologist is confident in ability of AI to undertake quantitative tasks and so is willing to trust the output and incorporate it into their decision making	=	Increased efficiency Increased accuracy
#2		Iterative design process with early user involvement Pathologists involved in decision to implement AI in their department and are given the opportunity to regularly feed into reviews of its benefits and costs	+	AI identifies regions of interest Explainable AI High usability Integration into workflow	Understanding the basis on which regions of interest have been identified, the pathologist is confident that all relevant regions of interest have been identified, reducing the percentage of slides, and the amount of the slide, they need to look at	=	Increased efficiency
#3	Smaller, nonspecialist departments	Iterative design process with early user involvement Pathologists involved in decision to implement AI in their department and are given the opportunity to regularly feed into reviews of its benefits and costs	+	Provision of ‘second opinion’ Explainable AI High usability Integration into workflow	Understanding the basis on which the opinion is made, the pathologist is willing to trust and accept the opinion	=	Increased accuracy
#4	Specialized team	Iterative design process with early user involvement Pathologists involved in decision to implement AI in their department and are given the opportunity to regularly feed into reviews of its benefits and costs	+	AI screens out negative cases Explainable AI High usability Integration into workflow	Understanding the basis on which positive cases have been identified, the team is confident that all positive cases have been identified, reducing the number of cases they need to look at	=	Reduced workload

### Participants

We interviewed pathologists from 5 National Health Service (NHS) hospital Trusts (healthcare organizations) in the north of England. This was a convenience sample, representing all healthcare organizations participating in the National Pathology Imaging Cooperative (a collaboration between the NHS, academia, and industry, leading the implementation of digital pathology [DP] within the NHS and developing AI tools for pathology; https://npic.ac.uk/), but included both teaching hospitals and district general hospitals. Together, these Trusts serve a population of over 2.3 million.

There is no consensus regarding how many interviews are necessary to provide an adequate understanding of attitudes and experiences within a particular setting but it partly depends on the range of participants to be included.^21^ We chose to undertake 25 interviews, allowing for a purposive sample of pathologists with variation in employing organization, years in practice, subspecialty, and experience of DP.

### Data collection

Semi-structured interviews were conducted by the first author using a realist technique called the teacher–learner cycle.^22^ Participants were asked questions that sought to elicit whether, and in what ways, the CMOcs fitted their perceptions and experiences (interview topic guide is provided in Supplementary File S1; further detail about the interviews is provided in Supplementary File S2). Because of Covid-19, almost all interviews were carried out by video or telephone call. Interviews were audio-recorded and transcribed verbatim.

### Analysis

Anonymized interview transcripts were entered into Nvivo 12. Analysis was undertaken using the framework approach^23^ (see Supplementary File S2 for further details). In preparing this paper, we have followed guidelines for reporting qualitative informatics research^24^ (see Supplementary File S4).

### Ethics

Ethical approval was granted by University of Leeds School of Healthcare Research Ethics Committee (ref: HREC 19-002). Approval to conduct the research was obtained from the UK Health Research Authority and each healthcare organization. Interviewees received no compensation for participating.

## RESULTS

We interviewed 20 pathology consultants (attending physicians), with between 1 and 30 years in role, and 5 pathology trainees (resident physicians). Fourteen interviewees identified as male, 11 as female. Nine consultants were generalists and 11 were specialists, covering gastrointestinal (GI), thoracic, gynecological, skin, breast, head and neck, renal, urological and neuropathology, in addition to nongynecological cytology. Nineteen used DP in some form, often for external quality assurance or referrals. Four consultants, all specialists, used DP for reporting cases. All trainees had DP experience. Interviews lasted between 33 and 85 min (mean duration: 56 min).

We organize our findings around, firstly, features AI should provide and, secondly, contextual factors to support uptake. Quotes illustrating the themes below are provided in Table 2.

**Table 2.** Illustrative quotes

Theme	Quote
Trust and explainable AI—pathology as a black box	“And in a way I wonder if we as pathologists do the same sort of thing? So especially the more experienced pathologists. They can look at a slide and say it has that sort of picture. Whereas as a junior pathologist, you'll go through in a very systematic fashion and describe each of the different layers and cell types and so on and so forth.” (5)
	“Take radiology, the clinicians always like to have a look at x-rays themselves. There’s a big difference there; yes, they read the radiology report, but they like to have a look at the results themselves as well. We work in a complete black box; they don’t question us, even though we’re working in highly subjective areas often.“ (8)
Trust and explainable AI—concern over AI tools that identify regions of interest	“Perhaps it would make you focus your attention more on those bits to the detriment of maybe not looking at all the rest quite so well or thoroughly like you normally would.” (9)
	“what I would like to do ideally would be to look at the case, you know, blind […] and then check. It would be a checking […] system rather than a sort of tell me what to do kind of thing.” (14)
High usability and workflow integration—preference for call up AI	“I think it would be helpful to use it selectively for me. When I have a really difficult renal tumour that I really don't know what it is, I would seek help of the AI and see. Because I would have narrowed down my possibilities, that might be helpful.” (16)
High usability and workflow integration—concern over incorporation of textual information	“Diagnosis is totally context-dependent and I don't think you can teach a machine how to interpret clinical context, it's too nebulous a concept actually. […] context is everything.” (25)
	“I’ve mentioned the variability in the lab. So, the staining, if it goes wrong then that would be tricky. Also, sometimes with lymph node screening […] you don’t get a full section […] would it say that something is negative because it hasn’t got anything in it? […] would it actually recognise that you might not be having […] an adequate section?” (15)
Specialists versus generalists—national screening programs	“I personally think it [AI] would probably be more useful in the national screening programmes because there you have a very tightly defined sample type. I think the one that obviously comes to mind is cervical screening. It's not done in many centres so you could standardise…it's high volume of something that's all the same, the techniques are incredibly tightly controlled and standardised across the country. And there's only about nine outcomes that that whole process produces. So to me, that would be where it would be the most useful” (25)
Specialists versus generalists—benefit for generalists	“You could argue that it would be more important in a district general hospital. If it was more general like ours then we probably wouldn't see as many cases of any particular type as a teaching hospital. And so a tool to help make sure that we were being accurate with our grading, for example, would be more useful in the district general. Again for the same reasons, probably generalists it would help out more on a more practical level.” (13)
User involvement—pathologist led implementation	“I think the most successful uses of AI in the first instance are going to be organic and are going to be things that come from suggestions within the department itself and the things that will address the particular needs of a particular department. So say […] you’re down two gynae consultants for a year on maternity leaves or sickness leaves, those pieces of AI that can, yeah, triage your cervical specimens or something, but that’s an obvious area that would be good. But […] I would think a blanket introduction from on high is likely to raise people’s suspicions.” (1)
Liability	“It would be a bit like getting a trainee to report a case and then not reviewing it, just signing it out. I would never do it. So even if you've got great trust in them because you think…because they're about to become a consultant and what have you, you would still look at the work, because you can't trust anyone that you're putting your name to. So, it's not worth it.” (7)
Evaluation and validation	“No, it may look good on paper, and you may publish your research, and show that this is much more standardised and much more accurate to a decimal point, but the real question is, does it make any difference to what the surgeon does, or what the clinician does, or what happens to the patient.” (23)
	“So I think the validating process is the most important thing, I think, which we found with the digital pathology as well, is that we validated very accurately and we identified the areas of errors very accurately, and we have put a system in place to deal with each of those inaccuracies or errors. And once you know that, then I think a pathologist would feel comfortable to still have the ownership of that diagnosis.” (18)
Resources	“I imagine… And it’s interesting, because I’ve said a general department as opposed to a teaching hospital but the teaching hospital tends to have more resources and finance through research and things. So it’s always the teaching hospitals that get all the new stuff first and then it dissipates out to us, which is the complete opposite of what I think would be needed in this. I think the resources and the money and the software would need to go out into the DGHs and that would be a big barrier, I think—money, and the backing really.” (9)

### Required features of AI

#### Trust and explainable AI

The literature suggested trust is needed for AI adoption in pathology and could be achieved with explainable AI.^25^ We did not ask interviewees explicitly about explainable AI, instead asking their thoughts on the black box nature of AI. While there were concerns among some interviewees, the majority did not see it as an issue so long as the tool was properly evaluated, demonstrating accuracy and usefulness. Several interviewees made the point that pathologists work as a black box; different pathologists have different ways of looking at samples which means a pathologist does not necessarily know what features a colleague assesses to come to a decision, and experienced pathologists may find it hard to explain their decision-making. The point was also made that other departments already see pathology as a black box; whereas clinicians will look at x-rays themselves, they do not look at pathology samples themselves and do not question the pathologist’s report. Some interviewees expressed reservations about explainable AI, on the basis that if AI is only allowed to work in a way that is explainable it may limit what AI is capable of.

Issues of trust varied according to functionality discussed. Interviewees’ comments suggested that, in line with our CMOcs, trust was not an issue when talking about simple quantification tasks. Similarly, there was general support for AI tools that identify regions of interest on a slide. Where there was reservation, it was not about the tool but how pathologists may respond to it, a concern pathologists may not look at an entire section properly, just the part the tool has highlighted, leading to small but important features being missed. Consequently, some interviewees wanted AI to act as a second reader, checking samples after a pathologist has seen them, with emphasis on increasing accuracy rather than improving efficiency. Also suggested was that usefulness of such a tool would vary according to subspecialty; in certain subspecialties (eg, breast and renal), lesions are generally well circumscribed, making them easy to spot, so here AI would provide limited benefit. However, in other subspecialties, lesions are often less well circumscribed (eg, prostate), so AI would provide greater benefit.

While AI tools supporting quantitative tasks and identifying regions of interest keep the diagnostic decision with the pathologist, trust becomes more of an issue for AI tools where the pathologist moves from being “in the loop” to “on the loop,”^26^ as with screening tools. The literature suggests AI may reduce workload among specialized teams, through screening out negative cases. Interviewees agreed it could provide a significant reduction in workload. There was greater acceptance of such tools for national cancer screening programs because of reduced task complexity, with little variation in what the tool is being asked to look at and a limited number of outcomes. Regardless, to accept such a tool, interviewees described needing to have confidence that it is as good as or better than a pathologist. They saw trust as being created through adequate evaluation and pathologists having time to become familiar with the tools before using them in routine practice, to allow them to become comfortable with AI and determine its strengths and weaknesses and usefulness. Similarly, comments about training suggested what was important was not just knowing how to use the system but understanding how AI works and knowing when use is appropriate.

#### High usability and workflow integration

Our CMOcs suggested high levels of usability would be important for acceptance of AI. The user interface was mentioned by several interviewees when asked what they needed to start using AI. Frequently mentioned was the need for an integrated system, aligning with our CMOcs that suggested the importance of workflow integration. This was considered important for efficiency and simplicity, with a general desire to simplify the rather complicated and cluttered series of systems pathologists use.

Interviews provided the opportunity to explore more fully what workflow integration may mean in practice. Discussion focused largely on whether AI should be automatic or only active when the pathologist calls it up. The pathologist remaining in charge but being helped by AI, as described above, was a widely expressed preference, leading to a preference for AI operating only when the pathologist selected to use it. Additionally, a number of interviewees anticipated using AI for specific tasks or when dealing with a difficult case, an aid they do not need most of the time.

The preference for call-up AI was also due to concern about whether automatic AI will incorporate contextual information appropriately. This included the patient context (age, gender, previous history or current treatments, etc) and the sample context (eg, quality of staining, adequacy of sample). However, the majority of interviewees expressed willingness to use automatic AI in at least some contexts, seeing this as more efficient. Several different uses of automatic AI were suggested, including screening out normal biopsies, ordering additional tests (such as immunohistochemistry) before the pathologist reviews the case, quantification, and checking cases for features the pathologist missed. However, for AI to be used automatically it would need to be thoroughly evaluated, a topic we return to when discussing contexts, and many interviewees expressed a desire to use it in a call-up manner initially.

### Contexts

#### Specialists versus generalists

Our CMOcs suggested benefits of AI will vary according to size and nature of the pathology department’s workload and the extent to which pathologists work collaboratively, with specialist centers benefitting from reduced workload, rather than increased accuracy. Our interviewees’ comments supported this; as described, there was support for using AI to screen out negative cases in national screening programs, reducing workload for specialists undertaking this work. Among the generalists we interviewed, there was concern about having AI undertake most simple cases, as this would leave them with just the difficult cases, increasing work intensity. Generalists diagnose a wide range of pathologies and apply a diverse set of grading rules; interviewees felt tools that supported this would enable generalists to deal with a greater range of work to a higher degree of accuracy.

#### User involvement

Our CMOcs suggested an iterative design process with early user involvement will support AI uptake. Some interviewees expressed a desire to influence AI development in an iterative process, feeding back weaknesses they identified. A widely expressed view was that pathologists should be involved as early as possible in design, to ensure the tool is suitable for as many pathologists as possible and addresses pressing needs, not just working where AI is strong. Also clear from interviewees’ comments was that what is useful varies by subspecialty.

Another contextual factor in our CMOcs was pathologist involvement in the decision to implement AI within their department. A widely held view was that AI implementation needs to be organic; the drive must come from within the department. However, interviewees also perceived external support as necessary, at hospital level and nationally, with support from organizations such as the Royal College of Pathologists (a professional membership organization for UK pathologists which oversees the training of pathologists; https://www.rcpath.org/) and England’s Department of Health & Social Care (the Government department responsible for health and social care policy). External support was perceived as needed in terms of providing financial resources but also having a role in training and in validation and standardization of systems.

#### Liability

In the literature and interviews, liability was a significant area of concern.^5^^,^^26^^,^^27^ Interviewees were largely comfortable with AI being used as an aid to decision making but concern about liability was expressed in relation to screening algorithms. Currently, when a pathologist is named on a report, they will always have reviewed the case. If a pathologist’s name will go on a report for a case that has been screened by AI, it is highly likely they would review the case, leading to time savings being lost.

Views about whether liability structures would have to change varied; while most felt liability should remain with the pathologist, some interviewees thought changes to liability would be necessary for AI to be accepted into normal working practice. Part of this will be clear legislation or guidance from professional bodies about where liability lies when using AI for diagnosis. Some interviewees saw evaluation of AI as an important step for reducing liability concerns.

#### Evaluation and validation

Interviewees frequently mentioned evaluation and validation unprompted. Algorithm validation refers to “offline” (not impacting clinical decision making) assessment of accuracy and safety,^9^^,^^28^ while clinical evaluation is concerned with safety and utility/effectiveness and takes place “live” in the clinical setting.^9^ However, interviewees used the terms evaluation and validation interchangeably. Interviewees’ comments suggested that, to establish trust, evaluation must demonstrate accuracy but also usefulness, showing it can improve accuracy or save time. Demonstrating usefulness includes demonstrating patient benefit, with several interviewees expressing the view that this will lead to AI adoption.

Interviewees described wanting to know how an AI tool was validated, particularly the data used to train and test the tool because of the bias this can introduce. Some interviewees described wanting to see AI evaluated in large scale clinical trials, to allow pathologists to determine how and where AI will perform well. They wanted to see AI evaluated in real-world situations, rather than being tested on samples where poor-quality slides have been removed and staining is highly consistent, and with evidence of patient outcomes.

Interviewees also stated the need for evaluation at departmental level, where each subspeciality tests the system, to identify limitations, allowing for creation of local guidance on AI use. Similarly, it was stated that pathologists would need to evaluate tools themselves, allowing them to become familiar with the system and involved in its implementation. These ideas fit with our CMOcs that pathologists should have the opportunity to feed into reviews of the benefits and costs of AI.

#### Resources

An additional issue raised was resources. Development and use of AI systems depends on pathologists viewing images on a computer. Of the 5 healthcare organizations where interviews were conducted, only one had DP infrastructure in place, but even here it was raised as being an issue. Several interviewees felt the existing IT infrastructure was not up to the standard required for DP or AI. Interviewees also stated that the NHS has a poor reputation for IT and change tends to be slow and unwieldy.

## DISCUSSION

We have described interviewees’ theories concerning what is needed for the uptake of AI in pathology. Table 3 summarizes our findings in the form of refined CMOcs, italics showing the refinements. The most significant change is the removal of explainable AI as a required feature. Instead, rigorous evaluation that demonstrates patient benefit will motivate pathologists to use AI and, when combined with opportunities for pathologists to assess the strengths and weaknesses of the tools, will give them the confidence to do so. Other changes relate to context: the need for adequate IT infrastructure and support from the hospital and national bodies. AI tools that highlight regions of interest will only provide benefit in specific subspecialties and the preference is for such tools to act as a “second reader,” so the benefit is increased accuracy rather than efficiency. General pathologists will benefit from tools that support grading while, in the context of national cancer screening programs, specialists will benefit from tools that screen out negative cases, but only with clear legislation or guidance regarding liability.

**Table 3.** Refined Context–Mechanism–Outcome configurations

	Who	Context	+	Mechanism		=	Outcome
				Resource	Response		
#1		Iterative design process with early user involvement *Rigorous evaluation with demonstration of patient benefit* Pathologists involved in decision to implement AI in their department and are given the opportunity to *assess its strengths and weaknesses* *Adequate IT infrastructure* *Support from hospital and national bodies*	+	AI undertakes quantitative tasks High usability Integration into workflow	*Perceiving benefits of AI for patients and understanding its capabilities, the pathologist* is willing to incorporate the output into their decision making	=	Increased efficiency Increased accuracy
#2	*Specific subspecialisms, for example, prostate*	Iterative design process with early user involvement *Rigorous evaluation with demonstration of patient benefit* Pathologists involved in decision to implement AI in their department and are given the opportunity to *assess its strengths and weaknesses* *Adequate IT infrastructure* *Support from hospital and national bodies*	+	AI identifies regions of interest Explainable AI High usability Integration into workflow, *acting as a second reader*	*Having already checked the slide,* the pathologist is confident that all relevant regions of interest have been *considered*	=	Increased *accuracy*
#3	Smaller, nonspecialist departments	Iterative design process with early user involvement *Rigorous evaluation with demonstration of patient benefit* Pathologists involved in decision to implement AI in their department and are given the opportunity to *assess its strengths and weaknesses* *Adequate IT infrastructure* *Support from hospital and national bodies*	+	*AI supports grading* Explainable AI High usability Integration into workflow	*Perceiving benefits of AI for patients and understanding its capabilities,* the pathologist is willing to *incorporate the output into their decision making*	=	Increased accuracy
#4	Specialized team	Iterative design process with early user involvement *Rigorous evaluation with demonstration of patient benefit* Pathologists involved in decision to implement AI in their department and are given the opportunity to *assess its strengths and weaknesses and costs* *Adequate IT infrastructure* *Clear legislation or guidance from professional bodies regarding liability* *National Cancer screening programs*	+	AI screens out negative cases Explainable AI High usability Integration into workflow	Confident that *the tool is as good as or better than a pathologist, the team is willing to use it,* reducing the number of cases they need to look at	=	Reduced workload

At present, scarce empirical evidence exists regarding factors necessary for integrating AI into clinical practice,^29^ although trust has been identified as an important challenge.^30–32^ While some authors have argued for the importance of system performance and AI being able to explain or justify its conclusions,^29^ for our interviewees, system performance alone was considered adequate for generating trust. Pathology and radiology are often considered similar fields, but a survey of radiologists suggests that while, like our interviewees, they place emphasis on evaluation, including evaluation using their own data, they also require algorithms to be understandable.^33^ This difference may be due to what our interviewees described as the “black box” nature of pathologist decision-making. Interviewees’ lack of concern about the black box nature of AI parallels the finding that patients value explainability of AI systems less in healthcare than other domains, especially when weighed against accuracy.^34^

In terms of functionality AI should provide, previous studies point to quantitative tasks such as counting mitoses and measurement of tumor margins^6^ or simple, routine, or repetitive tasks.^7^ This aligns with our findings; interviewees were less cautious about quantitative tasks and, while they were also willing to consider AI screening out negative cases, this was in the context of decisions with reduced complexity.

Concerns about liability are not unique to pathology.^4^^,^^35^ Like our interviewees, a survey of radiologist found the majority believed physicians should be liable when using AI tools, although 41% felt vendors should share at least some responsibility for solutions certified by the Food & Drug Administration (FDA).^33^ More generally it has been found that the majority of physicians and patients consider liability should rest with the physician, although physicians are more likely than patients to believe vendors and healthcare organizations should be liable.^36^ An analysis of FDA-approved machine learning (ML) decision support tools found nearly half were assistive, with statements emphasizing clinician responsibility for the final decision or limiting the extent to which the tool could be relied on.^37^ Given this, interviewees’ preference for AI to act as a second reader seems appropriate. A recent FDA letter to healthcare providers advised on use of an AI tool for radiology, stating that images not flagged by the system with suspected findings, that is potentially negative cases, still need to be interpreted, raising the question of whether the use of AI to screen out negative cases in cancer screening programs is a realistic prospect.^38^

### Implications for practice

For those designing AI tools for pathology, the CMOcs align with best practice for design of HIT: involving users in the design process and ensuring high usability and workflow integration. Our findings provide insight into what this means in the context of designing AI tools in pathology. Specifically, involving pathologists in the design process means ensuring the tools developed address real needs but those needs will vary according to subspecialty. This does not mean all tools need to be subspecialty specific; for example, a tool to examine lymph nodes for abnormalities could be useful across several subspecialties. However, it does suggest the need for more nuance when making proposals for pathology AI tools; it cannot be assumed that a tool that is perceived as useful in one subspecialty will be perceived as equally useful in other subspecialties. Practically, this means there is a need to gather perspectives on the potential benefits of proposed tools with pathologists from a range of subspecialties. This also has implications for the transferability of results of AI evaluations; pathologists considering the results of an evaluation need to carefully consider whether their subspecialty would likely obtain the same benefits.

While workflow integration is important, our findings suggest users must be given control over whether AI works automatically or only on demand. In terms of design, this does not simply mean providing the user with an easy way to turn off the AI tool; there is a need for customizable settings at individual pathologist level that provide the pathologist with control over both which recommendations and support they receive and when they receive them.

For healthcare organizations wanting to introduce AI in pathology, the well-recognized need to involve users in the decision to introduce technology applies,^39^ but our findings suggest this is not enough; time is needed for evaluation of tools at a departmental and individual level, so users can make their own assessment of strengths and limitations of the tools and create local guidance on use.

For those evaluating AI tools in pathology, our findings suggest that clinical trials of AI are needed, but these remain rare,^40^^,^^41^ with most FDA-approved ML clinical decision support tools having been approved via premarket notification.^37^ Ideally trials should use patient outcome measures but, given where pathology sits on the patient pathway, demonstrating benefit for patients is likely to be challenging. In DP, evaluations have largely been validation studies, examining concordance between a diagnosis made on glass and digital images.^42^ Consequently, there is a need for further research concerning the evidence required to satisfy pathologists regarding AI’s benefits. While our interviewees largely talked about increases in accuracy through AI use, they also pointed to ways in which AI could support workflow and thereby increase efficiency, such as automatically ordering additional tests. Adequate workforce numbers are essential for ensuring the care quality and safety and such efficiency improvements could help to address the shortage of pathologists.^43^

### Strengths and limitations

The strength of this research is that interview questions were based on analysis of existing literature. Using the literature-based CMOcs as a starting point generated detailed discussion of contextual factors likely to support integration of AI into pathology.

Interviews were undertaken in a convenience sample of 5 organizations in the north of England, raising questions regarding generalizability. However, the CMOcs explored in the interviews were derived from international literature and, as described, some findings echo those of studies undertaken elsewhere, suggesting the findings are not UK-specific. Additionally, a realist approach supports *theoretical generalization*^44^; we present CMOcs that can be empirically tested across different settings, potentially revealing additional contextual factors to be added.^8^ Interviewees did not have experience of AI and may have responded differently if they did; the CMOcs presented can be tested and refined as AI is introduced into pathology.

## CONCLUSION

Using a realist approach, we have captured pathologists’ perceptions of factors likely to support and constrain AI adoption in pathology, providing recommendations for design of AI tools and their implementation. Explainable AI is not needed for establishment of trust, but rather robust evaluation and the opportunity for pathologists to become familiar with AI. Designers need to involve pathologists in design. There should be user involvement in the decision to introduce AI, with the opportunity for evaluation at the departmental and individual level, but guidance from national organizations is also needed, including regarding issues of liability.

## Supplementary Material

ocac254_Supplementary_Data

## Data Availability

Interview transcripts will be kept until August 2025 and can be accessed by other researchers during this time, subject to the necessary ethics approvals being obtained. Requests for access to these data should be addressed to the corresponding author.
