# Supplementary material for: How, for whom, and in what contexts will artificial intelligence be adopted in pathology? A realist interview study
Source: J Am Med Inform Assoc. 2022 Dec 24;30(3):529–38. doi: 10.1093/jamia/ocac254 (PMC9933065; doi:10.1093/jamia/ocac254)
Supplement: ocac254_Supplementary_Data [file ocac254_supplementary_data.zip › ocac254_Supplementary_Data/Additional file 1 Interview topic guide.pdf]

How, for whom, and in what contexts will artificial intelligence be adopted in pathology? A realist interview study (King et al.)

Additional file 1: Interview topic guide

Interview process and questions

1. **Obtain consent**
2. **Introduction**

   As part of the roll out of digital pathology by NPIC (northern pathology imaging co-operative) we're undertaking some research into pathologists' perspectives about using artificial intelligence (AI) in their working practice. This isn't because the DP roll out includes any AI, but digital pathology allows the possibility of AI developing in the future. This interview should help us understand the contexts in which histopathologists think AI can provide benefit (e.g. for which types of decisions) and what they consider needs to be in place for AI to provide this benefit (e.g. how it should be integrated into the workflow). This will help inform the development of AI tools for histopathology and their introduction into practice.

3. **The interview**

   The interview has 3 sections to it; the first is some background questions that should help give a context to your answers. The second section covers questions relating to situations where you see AI being used or useful. Finally we have some questions about support needed for AI to be implemented which includes barriers that need to be overcome. As part of the interview, I'll describe ideas from the literature or from other interviews about how AI might support histopathology. What I'll ask you to do is, based on your experience, comment on and discuss these ideas, so that we can learn from your experience.

- **Background questions**
    - What is your role? (consultant/trainee, specialism)
    - How many years have you been in that role?
    - What's your experience of using digital pathology? (how much, in what contexts, perceived benefits/limitations)
- **Situations where you see AI being used and or useful**
    - In order to gauge how to pitch and introduce the interview could I ask how familiar are you with ideas about the role AI could play in histopathology? Could you give some examples?
        - *(If relevant) Where would you say you've got most of your information about AI from to date?*
- (if required) Before we launch into this section I just want to take a minute to introduce AI and explain why these questions are relevant to the NPIC project. Generating digital images of slides makes it possible to develop and start using computer systems as a diagnostic aid. Part of the NPIC project is to develop these systems, particularly AI systems. At its most fundamental, AI describes machines (so computers or computer programmes) that mimic cognitive functions that we usually associate with humans, such as learning and problem solving. In the context of pathology this could be a programme that has been trained to recognise patterns in images,

such as finding cancer in lymph nodes. The questions that we are asking are the possibility of using AI in the future rather than digital pathology now.

- One idea is that AI can augment the decision making of histopathologists, so it's not making the decision but it's supporting you in making the decision. For example, it could highlight areas suspicious for malignancy in a biopsy. What are your thoughts about that?
- Are there particular types of cases or decisions where you think this would be beneficial?
    - *Could you explain a bit more? What is it about this type of case/decision that makes it particularly suitable?*
- Another idea is that AI could have a greater role in decision making, where AI makes the decision. For example, it has been suggested that AI could be used for providing the mitotic count component of a tumour grade. What are your thoughts about that?
- Are there particular types of decisions where you think this would be beneficial?
    - *Could you explain a bit more? What is it about this type of case/decision that makes it particularly suitable?*
- One possible use of AI is for it to remove easy, routine and mundane tasks (triaging simple benign cases eg. negative appendix or gallbladder). Do you think this would be useful?
    - *Why do you think that?*
    - *What contexts do you see it being applicable?*
    - *Can you see any downsides to using AI this way? Are there particular situations in which these downsides might occur?*
- Another use of AI is as an aid for difficult tasks (lymph node screening, prostate carcinoma grading). Would this be something you'd be willing to use?
    - *Could you explain your reasoning?*
    - *What contexts do you see it being applicable?*
    - *Can you see any downsides to using AI this way?*
- It's been suggested that AI could speed up workflows through a range of means such as removing simple cases or highlighting areas of interest. Do you think improved workflows would provide any benefit to you?
    - *Could you expend on that, what are your reasons?*
- AI could also improve the accuracy of a diagnosis through more reproducible measurements and grading decisions. Would this benefit you?
    - *Why would increased accuracy benefit you?*
- Do you think AI would be more beneficial in a teaching hospital or district general hospital (large and specialist vs small and general)?
    - *Could you explain a bit more?*
- Do you think there are particular groups of histopathologists that would benefit? (trainees vs consultants, specialists vs generalists)

    - *What is it that leads you to those conclusions?*
- Do you think AI should enhance a person's strengths? Or should it strengthen people's weaknesses? We're looking to try and tease out where who AI would be best for.
    - *What is it that makes you think that?*

- **Support needed for AI to be implemented**
    - What would need to be in place for you to begin using AI in your work?
    - And what might get in the way of you using AI in your work?
    - Do you think there's a group most likely to drive the uptake of AI within pathology? Why?
        - *Will it have to be from within the department or could an external body (the hospital management, government, professional body) drive it?*
    - By taking the initiative on AI implementation do you think pathologists can remain in control of what AI does?
        - *(Why) would that be useful?*
    - Some pathologists would like AI to be a tool that is called upon when required; for example an IHC scoring tool or a grading tool that is selected once the pathologist has determined that it is necessary. Would you agree with this?
        - *If yes, could you explain a bit more? Why do you think it is important? How do you think this would make a difference?*
        - *Would there have to be clear policies over the context in which it's used?*
    - Others have suggested it's about making sure that AI is integrated into the workflow of the histopathologist with AI always active. For example a tool that automatically highlights areas of potential abnormality. Would you agree with this?
        - *If yes, could you explain a bit more? Why do you think it is important? How do you think this would make a difference?*
    - Pathologists have a reputation of being resistant to change, yet every interview we've done so far has shown that pathology has changed significantly and that pathologists are highly interested in providing a service that has the most benefit to patients. Why do you think that pathologists have this reputation of being resistant to change?
    - Some people have suggested that AI will lead to pathologists being less involved in diagnosis and more involved in treatment selection and patient management. Would you be happy being less involved in the diagnosis but more involved in treatment decisions/giving more advice to clinicians?
    - The introduction of new technology and systems can cause changes to a professional's established role. This can cause conflict as changes are introduced. Can you see any conflicts arising from the implementation of AI, both within histopathology and with other departments/organisations?
    - Do you think AI will alter the perception of pathology by other departments?

    - Why do you think that would be the case?

- Pathologists are currently responsible for the diagnosis and decisions that they make. Increased use of AI may shift decision making away from humans. Some people have suggested that pathologists' concerns about liability will make them reluctant to use AI, so that there is reduced uptake of AI. Would you agree with this?
    - *Are you comfortable with this?*
- Do you think liability structures should change if AI is introduced?
    - *How should it change, should companied bare some of the liability?*
- Would concerns about liability make you less likely to use AI tools?
- Do you think there will ever be a situation where AI makes diagnoses by itself?
    - *If no, will it be patient objections or pathologist objections that cause this?*
- A lot of research has been done in other fields about unintended consequences of automation. One issue that comes up regularly is how automation de-skills the workforce, because people are less involved in the work, and yet people have to remain in charge of the system. Can you see a situation like this being a problem in pathology?
    - *Could you explain why you think that?*
    - *What could be done to avoid it?*
- Some algorithms, particularly the most advanced ones, are not easily interpretable; they don't explicitly describe the features that they assess when making a decision. What are your thoughts on this?
    - *Could you elaborate in that?*
    - *There are medicines that are used where the mechanisms aren't know (paracetamol, GA), is this a similar situation?*

- **Additional questions**
    - Improving workflow would allow pathology to maintain its professional boundaries but would not alter the pathologist's abilities. Improving diagnostic accuracy would not necessarily maintain professional boundaries but may mean that pathology expends into new areas such as patient management and treatment selection. Do these scenarios sound possible?
        - *Which of them do you think is the most significant it determining the future direction of pathology as a speciality?*
    - It has been suggested that AI could enhance a person's ability whilst at the same time undermining their status. For example, with AI you get through more cases making more accurate and treatment determining diagnoses but the perception is that it's the algorithm not you. Do you think this would be possible in pathology?
        - *Would there be any negative consequences from this?*

- As AI improves it's been suggested that pathology and radiology (and possibly genetics) merge to form a new speciality of information experts (synthesising advice for clinicians from the information that these different sources provide). Can you see this happening?
    - *Can you give your reasoning for that?*
    - *If it did happen how would you feel about it?*
- It's often said that fundamental pathology practice has changed very little over the years. Do you agree this this?
    - *What makes you think this?*
    - *If yes, will this continue to be the case?*
- What changes do you think will take place during the next few (5) years?
- How do you think (the changes above) will affect your role as a pathologist?
    - *If they expect change to their role, how do you feel about your role changing?*
- Where would you say you've got most of your information about AI from to date?
- What would you say is your overall view of AI?
- Do you think that AI will improve your working life?
    - *How (if not elaborated on)?*
- Are there any other concerns that you have or know about in relation to using AI?
    - *If not mentioned follow up with: What is your understanding of bias in AI algorithms*
