# Supplementary material for: How, for whom, and in what contexts will artificial intelligence be adopted in pathology? A realist interview study
Source: J Am Med Inform Assoc. 2022 Dec 24;30(3):529–38. doi: 10.1093/jamia/ocac254 (PMC9933065; doi:10.1093/jamia/ocac254)
Supplement: ocac254_Supplementary_Data [file ocac254_supplementary_data.zip › ocac254_Supplementary_Data/Additional file 2 Methodological appendix.pdf]

How, for whom, and in what contexts will artificial intelligence be adopted in pathology? A realist interview study (King et al.)

Additional file 2: Methodological appendix

Data collection

Interviews in this study were conducted by the first author (HK) using a realist technique called the teacher-learner cycle.[1] In teacher-learner cycle interviews, participants are presented with the literature-based theories and asked to reflect on whether, and in what ways, those theories fit with their own perceptions and experiences and to refine or modify these ideas accordingly (the idea being that the researcher first teaches the interviewee about their theories, and then the interviewee teaches the researcher through helping them to refine their theories). While such an approach is very different to a typical qualitative interview where the interviewer is expected to put aside any preconceptions or assumptions, realists argue that the interviewer always has their own theories when going into an interview, which influences the questions they ask and how they ask them, and similarly the interviewee always has their own ideas about what the interviewer is interested in, which influences the answers they provide. Therefore, in theory-driven research, a more productive approach is to use the interview as a vehicle for enabling participants to revise and expand these theories.

Early in the interview, we asked participants how familiar they were with ideas about the role AI could play in pathology and, where necessary, provided a description of AI and how it might be applied in pathology. Many questions focused on the functionality AI might provide as captured in the CMOcs, e.g. identifying regions of interest, exploring whether they thought it would be useful and in what contexts.

HK was new to qualitative research but received training and guidance from RR, who is an experienced qualitative researcher with expertise in realist methods. HK led the literature review that provided the basis for the interview study and so already had familiarity with realist methods. HK knew two of the interviewees prior to undertaking the interviews, but he had no prior relationship with the other 23 interviewees.

Analysis

Anonymized interview transcripts were entered into Nvivo 12. Framework analysis was used,[23] due to its systematic nature and ability to provide a clear audit trail.[24] Once 20 interviews had been undertaken, one author (HK) listened to the recordings and developed a thematic framework. All codes were inductively derived but, following the realist strategy, sought to capture different contexts, mechanisms, and outcomes (see Additional File 3). A second author (RR) read transcripts of a sample (three) of interviews to identify additional codes. HK then used the codes to index the data. Coded data were summarized by HK in a matrix, enabling all authors to identify patterns in the data and offer their perspectives.[24] Following discussion of the matrix, HK produced narrative summaries of each of the identified contexts, mechanisms, and outcomes, with quotations to illustrate findings, which were discussed by the authors. RR compared the narratives to the CMOcs from the literature review, adding to and refining the CMOcs where appropriate, returning to the matrix and original data where necessary.

References

1. Pawson R. Theorizing the Interview. The British Journal of Sociology 1996;**47**(2):295-314
