# Supplementary material for: How, for whom, and in what contexts will artificial intelligence be adopted in pathology? A realist interview study
Source: J Am Med Inform Assoc. 2022 Dec 24;30(3):529–38. doi: 10.1093/jamia/ocac254 (PMC9933065; doi:10.1093/jamia/ocac254)
Supplement: ocac254_Supplementary_Data [file ocac254_supplementary_data.zip › ocac254_Supplementary_Data/Additional file 3 Thematic framework.pdf]

How, for whom, and in what contexts will artificial intelligence be adopted in pathology? A realist interview study (King et al.)

Additional file 3: Thematic framework

Background
- Specialty: descriptions of interviewee's role and sub-specialism, e.g. trainee, breast pathologist
- Years in role: number of years as a consultant; for trainees, stage of training
- Experience with digital pathology: interviewees' descriptions of experience of using digital pathology and in what context, e.g. for research, quality assurance etc.

Context
- Conflict-resistance: interviewees' perspectives on whether there will be resistance to use of AI and whether it could be a source of conflict, both within the department and with other departments
- Implementation-who etc: interviewees' perspectives on how AI should be introduced
- Infrastructure: interviewees' comment about the need for adequate infrastructure
- Interface: interviewees' comments about the importance of the interface of an AI tool
- Money: interviewees' comments about the cost of AI, including cost as a potential barrier and concerns about cost and value for money
- Time: interviewees' comments about the need for adequate time to become familiar with AI tools
- Training: interviewees' comments about the need for training to use AI

Outcomes - Benefits
- Accuracy-consistency: interviewees' comments about the potential for AI to increase accuracy and consistency
- Computer vs human: interviewees' comments about the advantages of AI over pathologists, e.g. doesn't get tired
- Learning algorithms: interviewees' comments about the advantages of algorithms that learn
- Time saving: interviewees' comments about how and in what contexts AI will provide benefits in efficiency

Outcomes - Concerns
- Altered scales: interviewees' concerns about the potential for AI to change grade boundaries
- Black box: interviews' attitudes to AI as a black box, both positive and negative
- De-skilling: interviewees' concerns about the potential for pathologists to over-rely on AI and/or become deskilled through use of AI
- Impact on training: interviewees' perspectives on potential role of AI in pathologist training and its impacts, both positive and negative, on pathologist training
- Liability: interviewees' concerns about liability and comments about where liability should lie when using AI
- Loss of trust: interviewees' comments about the trust placed in pathologists and whether other clinical areas and patients would place the same trust in AI
- Power of suggestion: interviewees' concerns about the potential for AI to negatively influence

pathologist decision making, e.g. focusing on particular areas of a slide, and not wanting their decision to be biased by the AI recommendation
- Work intensity: interviewees' concerns regarding intellectual intensity of work if 'easy' tasks are undertaken by AI

How AI is used
- Automatic: interviewees' attitudes to the idea of automatic AI
- Call up: interviewees' attitudes to the idea of on-demand AI
- Primary: interviewees' attitudes towards using AI as a primary reader
- Secondary: interviewees' attitudes towards using AI as a second opinion

Role of pathologists
- Reputational loss: interviewees' concerns about the possible negative impact of AI on perception of pathology as a profession

Validation: interviewees' comments about how AI should be evaluated

What AI does
- Counting-measuring: interviewees' attitudes towards the use of AI for counting and measuring tasks
- Diagnosing: interviewees' attitudes towards the use of AI for diagnosis
- Screening-highlighting: interviewees' attitudes towards the use of AI for screening out negative cases and highlighting regions of interest

Where AI will be used: interviewees' perspectives on settings where AI will provide greatest value, e.g. teaching hospital vs district general hospital, for generalists vs specialists
