# Supplementary material for: How, for whom, and in what contexts will artificial intelligence be adopted in pathology? A realist interview study
Source: J Am Med Inform Assoc. 2022 Dec 24;30(3):529–38. doi: 10.1093/jamia/ocac254 (PMC9933065; doi:10.1093/jamia/ocac254)
Supplement: ocac254_Supplementary_Data [file ocac254_supplementary_data.zip › ocac254_Supplementary_Data/Additional file 4 Adherence to guidance for qualitative research.pdf]

How, for whom, and in what contexts will artificial intelligence be adopted in pathology? A realist interview study (King et al.)

Additional file 4: Adherence to Ancker et al.'s guidance for reporting qualitative research in informatics

Guidance	Reported?	Page number
1. Theory		
a. Cite theory appropriate to the topic being studied if applicable	Yes – presentation of tentative CMOs	p.6-8
2. Research question and study design		
a. State the research question	Yes	p.3
b. State the study design and methodological perspective of the research	Yes	p.4
3. Sampling		
a. Describe the sampling approach	Yes	p.8
b. Describe any approaches to ensure the inclusion of people from marginalized or underserved groups	No – not applicable	
c. Report and justify the sample size	Yes	p.8
d. If using saturation to determine sample size, report what type of saturation was used, and how saturation was assessed*	No – not applicable	
4. Data collection		
a. Report how data were collected	Yes	p.8 and Additional File 2
b. Report any methods for reducing bias in data collection and analysis*	Yes	Additional File 2
5. Data analysis		
a. Describe data analysis methods, with appropriate citations*	Yes	p.9
i. For deductive analysis, report how the theory was used in data collection and analysis*	Not applicable	
ii. For inductive analysis, report how the steps of inductive analysis were done*	Yes	Additional File 2
iii. For theory development, report how categories were developed*	Not applicable	
b. Describe any methods for improving the dependability of coding*	Yes	Additional File 2

c. Report any measures for improving the credibility of findings or verifying interpretations*	Yes	Additional File 2
6. Results		
a. Report sample size and characteristics of participants	Yes	p.9
b. Support thematic findings with extracts, quotes, images, or observations	Yes	p.15-17
c. Provide synthesis and interpretation	Yes	p.9-15
7. Discussion		
a. Describe assumptions of the research and details of setting and context to illustrate transferability of findings	Yes	p.23
b. Describe relationship of findings, or new theory developed in the study, to existing theory	Yes – description of refined CMOs	p.17-21
c. Report limitations	Yes	p.23-24
**Elements with an asterisk may need to be elaborated in an appendix to avoid lengthening the manuscript.*		
